# Chilling Does Not Affect the Functionality of Intracellular Calcium Stores in Viable Boar Sperm During Liquid Preservation

**DOI:** 10.3390/ijms27031248

**Published:** 2026-01-27

**Authors:** Doanh H. Bui, Anne-Marie Luther, Dagmar Waberski, Heiko Henning

**Affiliations:** 1 Unit for Reproductive Medicine, Clinic for Swine and Small Ruminants, University of Veterinary Medicine, 30559 Hannover, Germany; bhdoanh@gmail.com (D.H.B.); anne-marie.luther@tiho-hannover.de (A.-M.L.); heiko.henning@fli.de (H.H.); 2 Faculty of Animal Science, Vietnam National University of Agriculture, Hanoi 12406, Vietnam; 3 Institute of Farm Animal Genetics, Friedrich-Loeffler-Institut, 31535 Neustadt, Germany

**Keywords:** boar semen, cold shock, thimerosal, semen preservation, hypothermic storage

## Abstract

In mammalian sperm, the regulation of intracellular calcium (Ca^2+^) is essential for fertility. Semen processing for assisted reproduction may disturb Ca^2+^ homeostasis. This study aimed to investigate whether chilling boar sperm to 5 °C and subsequent storage affect the function of intracellular Ca^2+^ stores. Semen was stored in BTS-extender at 5 °C or 17 °C (control) for up to five days. Fluo-4/AM-loaded aliquots were incubated in Ca^2+^-free Tyrode’s medium at 38 °C. Sperm preserved at 17 °C had higher free intracellular Ca^2+^ levels compared with those stored at 5 °C (*p* < 0.05). However, there was no difference between storage groups in Ca^2+^ levels during incubation at 38 °C. Thimerosal, a sensitizer of Ca^2+^ channels, was added, and changes in the free intracellular Ca^2+^ concentration were monitored in viable acrosome-intact sperm by continuous flow cytometry. There was no effect of storage temperature on the kinetic response to thimerosal at days 1 and 3. At day 5, the relative increase in Ca^2+^ was higher in 5 °C-stored samples after 3 min of incubation. At 60 and 120 min of incubation, the thimerosal response was no longer influenced by the storage temperature or storage duration. In conclusion, chilling and storage do not affect the release dynamics of free Ca^2+^ from intracellular stores in viable boar sperm after rewarming.

## 1. Introduction

Boar semen is typically stored in a liquid state at 16 to 18 °C to avoid any chilling injury associated with lower temperatures. Triggered by the increasing prevalence of antimicrobial resistance arising from the routine use of antibiotic additives in semen extenders, cold storage of boar semen at 5 °C has recently been introduced as a preservation method that permits the omission of antibiotics. Despite the high performance of preserved semen in vitro and in vivo (reviewed by Waberski & Luther, [1]), there remain concerns that chilling injury could harm boar spermatozoa, particularly with subsequent long-term storage.

Pig breeding is an economically important agribusiness sector [2], in which even minor, sublethal sperm damage may compromise the performance and profitability of traded semen. Consequently, there is a need to further explore possible subtle effects of chilling on aspects of sperm function that are essential for fertilization.

A key component of sperm function is the ability to regulate free cytosolic calcium (Ca^2+^). Calcium serves as a crucial second messenger in physiological processes in mature sperm (reviewed in Mata-Martinez, et al. [3]). The capability for intracellular Ca^2+^ storage and release is central to capacitation, hyperactivation, and the acrosome reaction [4,5,6]. Sperm organelles and membranous compartments that can serve as calcium reservoirs include the acrosome, mitochondria, and calreticulin-containing vesicles of the redundant nuclear envelope [5,7]. All of those are present in boar spermatozoa, like in the sperm of other mammalian species.

To elicit the appropriate spatio-temporal oscillations in cytosolic Ca^2+^ levels, free intracellular Ca^2+^ is tightly regulated in sperm. The regulatory machinery includes a variety of Ca^2+^ transporters, e.g., Ca^2+^-ATPases and Na^+^-Ca^2+^ exchangers [8,9]; Ca^2+^ channels in the plasma membrane [10]; Ca^2+^-binding structures in the cytoplasm (calmodulin; [11]); and intracellular Ca^2+^ stores in the acrosome, the redundant nuclear envelope, and mitochondria [11,12,13].

These stores play a central role in regulating intracellular Ca^2+^ concentration and are operated mainly by sarcoplasmic–endoplasmic reticulum ATPases that are located in the acrosome and midpiece of boar spermatozoa [9] and by channels that mobilize stored Ca^2+^, such as those gated by inositol 1,4,5-trisphosphate receptors (IP_3_R) and ryanodine receptors (RyR) [12,13,14] (as reviewed by Mata-Martinez, et al. [3]).

It is well known that semen freezing or cooling followed by storage at temperatures above 0 °C disturbs intracellular Ca^2+^ homeostasis, Ca^2+^ signaling, and ultimately sperm function [15,16,17]. These phenomena are mainly attributed to increased leakiness to Ca^2+^ ions at the plasma membrane, allowing entry of extracellular Ca^2+^. The effects of cooling on the regulation of cytosolic Ca^2+^ levels by intracellular stores are less clear, particularly in sperm that survive chilling stress.

Therefore, the aim of this study was to investigate whether chilling and storage impair the functionality of intracellular Ca^2+^ stores in viable acrosome-intact boar spermatozoa after rewarming. To this end, a moderate chilling injury was provoked by the rapid cooling of semen in a short-term extender before storage at 5 °C, whereas samples stored at 17 °C served as the control. The effects of chilling and storage on basal, free intracellular Ca^2+^ levels and on the kinetic response of viable acrosome-intact sperm to thimerosal (sodium ethylmercurithiosalicylate), a sensitizer of IP_3_R- and RyR-gated Ca^2+^ channels, were studied.

## 2. Results

### 2.1. Effects of Storage Temperature and Time on Motility Parameters, Viability, and Acrosome Integrity

Motility (% total motile sperm) was significantly influenced by the semen storage temperature (*p* < 0.05, Table 1). On day 1 (d 1) of storage, motility in samples stored at 5 °C was significantly lower compared with the RT samples (d 0) and samples stored at 17 °C (*p* < 0.05). Samples stored at 17 °C did not show a decline in motility compared to the RT samples until day 3 of storage. Storage time had no effect on sperm motility at either temperature (*p* > 0.05). The mean values for velocity, linearity, beat-cross frequency, and amplitude of lateral head displacement of progressively motile sperm were not influenced by the storage temperature or time (*p* > 0.05). A storage temperature of 5 °C reduced the viable acrosome-intact sperm population on day 1 of storage compared with the RT samples (d 0) and samples stored at 17 °C (*p* < 0.05). Prolonged storage up to day 5 (d 5) had no effect on the percentage of viable acrosome-intact sperm stored at 5 °C or 17 °C (Table 1). In summary, semen stored in BTS at 5 °C showed a moderate reduction in sperm motility and membrane integrity, indicating a suitable condition for subsequent investigation of chilling injury in this study.

### 2.2. Effect of Storage Temperature and Time on Baseline Free Intracellular Ca^2+^ Levels During Incubation at 38 °C

**Figure 1:**
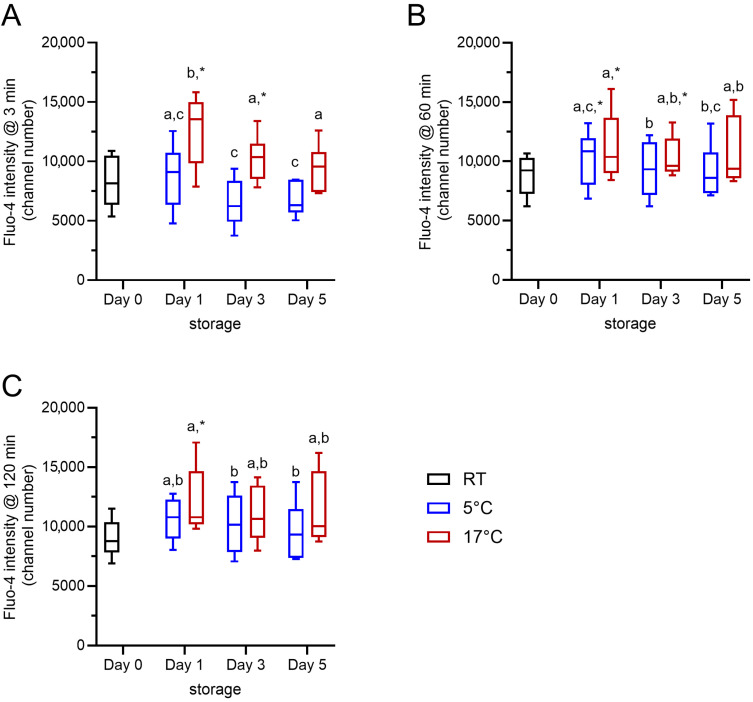
Intracellular Ca^2+^ levels for samples at RT (day 0) and subsequently stored at 17 °C (red) or 5 °C (blue) for up to 5 days (n = 6 boars). Baseline values of the average fluorescence intensity for Fluo-4, i.e., the free intracellular Ca^2+^ level, in viable acrosome-intact sperm after 3 min (**A**), 60 min (**B**), and 120 min (**C**) incubation at 38 °C have been plotted. Storage temperature (17 °C vs. 5 °C) and storage time (day 1 vs. day 3 vs. day 5) had a significant impact on the intracellular Ca^2+^ levels (*p* < 0.05). Different small letters (a–c) indicate significant differences (*p* < 0.05). An asterisk indicates significant differences between a given storage time by temperature combination and the reference point, i.e., the sample on the day of dilution (day 0; *p* < 0.05).

Changes in the free intracellular Ca^2+^ levels due to the storage temperature and storage time were evaluated from baseline values for the fluorescence intensity of Fluo-4 in control samples after 3, 60, and 120 min of incubation at 38 °C in Ca^2+^-free Tyrode’s medium (Figure 1). The gating strategy considered exclusively viable spermatozoa with intact acrosomes. The storage temperature had a significant effect (F(1, 5) = 31.240, *p* < 0.01) on the free intracellular Ca^2+^ levels in viable acrosome-intact boar sperm, and the storage time also had a significant effect (F(2, 10) = 10.538, *p* < 0.01). At 3 min, samples stored at 17 °C had consistently higher free intracellular Ca^2+^ levels than those samples stored at 5 °C (*p* < 0.05). The effects of storage temperature (F(1, 5) = 7.882, *p* < 0.05) and storage time (F(2, 10) = 4.221, *p* < 0.05) were weaker after incubation for 60 min and absent after 120 min (*p* > 0.05). At 60 and 120 min of incubation at 38 °C, there was no difference in intracellular Ca^2+^ levels between the samples stored at 17 °C and 5 °C. In summary, viable acrosome-intact boar spermatozoa are able to maintain low intracellular free Ca^2+^ after chilling. During rewarming to body temperature, intracellular Ca^2+^ levels are similarly regulated in chilled and control sperm.

### 2.3. Dose–Response Effect of Thimerosal on Intracellular Ca^2+^ Concentration

The addition of thimerosal induced a rapid and dose-dependent rise in the free intracellular Ca^2+^ concentration in all samples, whereas no change was observed in the control samples. Changes in intracellular Ca^2+^ levels were reported as relative changes, with the baseline value serving as the reference point for each measurement. The gating strategy considered exclusively viable spermatozoa with intact acrosomes.

**Figure 2:**
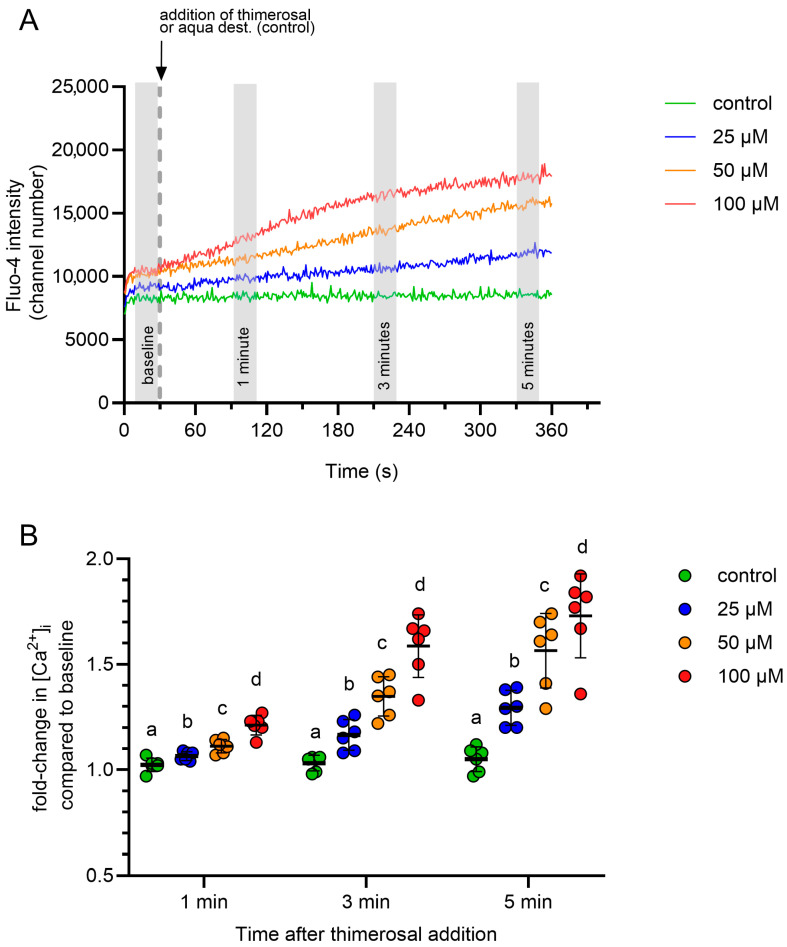
(**A**) Line graph illustrating the change in average fluorescence intensity for Fluo-4 in viable acrosome-intact spermatozoa to increasing concentrations of thimerosal or a solvent control (aqua dest.). Each line is the average of data from six independent measurements (n = 6 boars). Average fluorescence intensities for Fluo-4 for each boar were calculated for 20 s intervals directly before the addition of thimerosal (baseline), and 1 min, 3 min, and 5 min after the addition of thimerosal (shaded areas). (**B**) Relative changes in fluorescence intensity after the addition of thimerosal or aqua dest. (control) were calculated in relation to the baseline values of each curve. Different small letters (a–d) indicate significant differences between treatments at a given time point (*p* < 0.05; mean ± SD, n = 6 boars).

Representative dose–response curves are shown in Figure 2A. A concentration of 100 µM thimerosal was selected for subsequent experiments because it produced the most pronounced changes at all evaluated time points (1, 3, and 5 min) after addition (Figure 2B). In summary, a thimerosal concentration of 100 µM was identified for studies of Ca^2+^ release from intracellular stores.

### 2.4. Localization of Free Intracellular Ca^2+^ Signals

**Figure 3:**
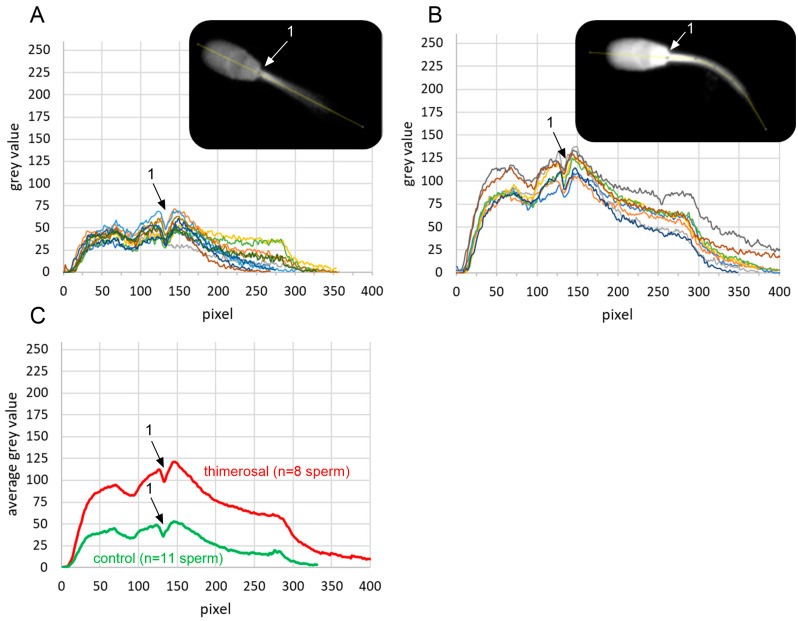
Localization and quantification of free intracellular calcium levels in viable boar spermatozoa on the day of semen collection, before and after exposure to thimerosal. Fluo-4 signal intensities in viable, i.e., propidium iodide-negative spermatozoa, were quantified in the longitudinal axis of the sperm head and anterior midpiece (yellow line in gray-scale images inserted in (**A**,**B**)) in ImageJ. Intensity values were plotted for control (aqua dest.; (**A**); n = 11 spermatozoa) and exposure to 100 µM thimerosal for 5 min ((**B**); n = 8 spermatozoa). Each colored line represents the intensity profile for an individual spermatozoon. Intensity profiles were aligned based on a characteristic local minimum in fluorescence intensity at the junction between the sperm head and tail, indicated by position ‘1’ in the images and plots. Average gray values for both groups are shown in (**C**).

The localization and quantification of signal intensities in viable acrosome-intact sperm treated with 100 µM thimerosal, and the control (aqua dest.), are shown in Figure 3A,B. Thimerosal increased the overall fluorescence intensity of Fluo-4 compared to the control condition (Figure 3C). Calcium signals were visible throughout the whole midpiece and principal piece during live imaging—the highest signal intensities after thimerosal application were observed on the head base and the proximal midpiece. Due to an out-of-focus position of the tail during image acquisition, calcium signals were not visualized in the distal half of the midpiece and alongside the principal and end piece of the sperm tail.

### 2.5. Effects of Storage Temperature and Time on Thimerosal-Induced Release of Ca^2+^ from Intracellular Stores

**Figure 4:**
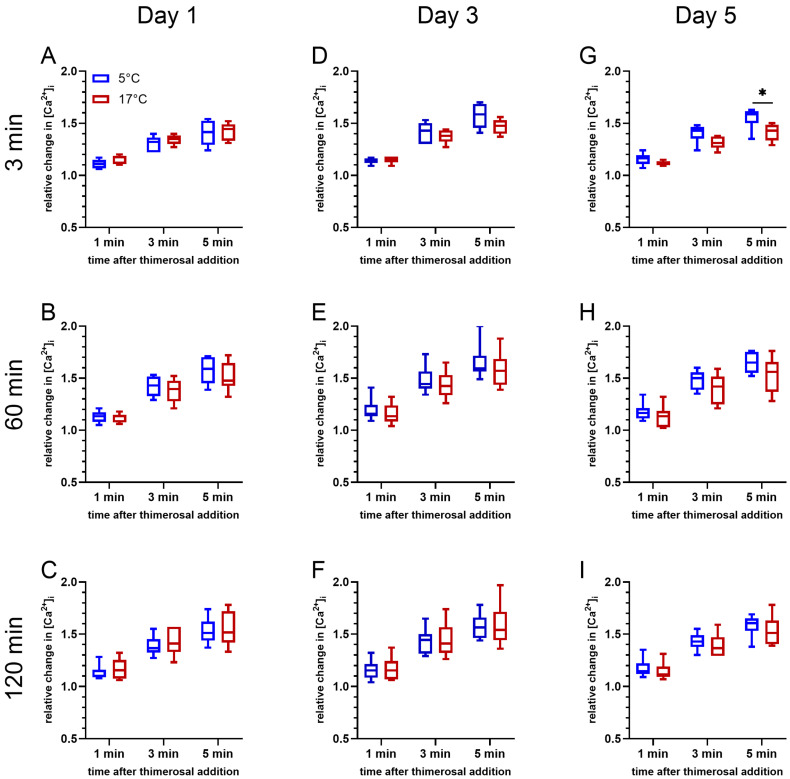
Changes in free intracellular Ca^2+^ levels in viable acrosome-intact spermatozoa after 3 min (**A**,**D**,**G**), 60 min (**B**,**E**,**H**), and 120 min (**C**,**F**,**I**) of incubation at 38 °C for samples stored at 17 °C or 5 °C for up to five days (n = 6 boars). Changes in fluorescence intensity for Fluo-4 (F1) in relation to a reference point (F0), i.e., the baseline fluorescence intensities before addition of 100 µM thimerosal have been calculated (relative intracellular Ca^2+^ level = F1/F0). An asterisk indicates significant differences between the samples stored at different temperatures (*p* < 0.05).

An effect of storage temperature on Ca^2+^ release from intracellular stores induced by 100 µM thimerosal was absent on day 1 (d 1) of storage (*p* > 0.05; Figure 4A–C). At all timepoints, the gating strategy considered exclusively viable spermatozoa with intact acrosomes. A significant effect of storage temperature was noted on day 5 (d5) of storage after 5 min of incubation at 38 °C (F(1, 5) = 6.691, *p* < 0.05; Figure 4G). The relative change in [Ca^2+^]_i_ for sperm stored at 5 °C tended to be higher 1 min (*p* = 0.060) and 3 min (*p* = 0.080) after the thimerosal addition, and was significantly higher 5 min after the thimerosal addition. A similar trend was observed after 60 min of incubation at 38 °C on day 3 (F(1, 5) = 5.955, *p* = 0.059; Figure 4E) and day 5 (F(1, 5) = 4.497, *p* = 0.087; Figure 4H). At both time points, samples stored at 5 °C tended to show a greater amplitude in the initial increase in intracellular Ca^2+^ levels, i.e., 1 min after thimerosal addition (Figure 4E: *p* = 0.079; Figure 4H: *p* = 0.095). In addition, the maximum increase in intracellular Ca^2+^ levels at 3 min (Figure 4E: *p* = 0.066; Figure 4H: *p* = 0.103) and 5 min (Figure 4E: *p* = 0.064; Figure 4H: *p* = 0.081) after the addition of thimerosal tended to be higher. After 120 min of incubation, changes in free intracellular Ca^2+^ levels showed non-significant differences between the samples stored at 17 °C and at 5 °C, regardless of the semen storage time (Figure 4C,F,I).

The aforementioned data focus on evaluating the effects of the storage temperature and storage time. However, it should be noted that Ca^2+^-release dynamics from intracellular stores after the thimerosal addition consistently showed a lower amplitude for the samples stored at 17 °C after 3 min of pre-incubation on all days of storage (d 1, d 3, and d 5), compared with freshly diluted samples on the day of semen collection (d 0; Supplemental Figure S1). For samples stored at 5 °C, this was evident only at d 1. After 60 or 120 min of pre-incubation, no differences in release dynamics between the stored and fresh samples were evident (Supplemental Figures S2 and S3). In summary, after chilling and storage, viable acrosome-intact sperm maintained their ability for Ca^2+^ release from intracellular stores after rewarming to body temperature.

## 3. Discussion

Understanding the functional alterations in viable spermatozoa is essential for elucidating the impact of preservation-related stressors on sperm fertilizing capacity. The present study shows that chilling and storage do not have a pronounced effect on the kinetics of modulator-induced Ca^2+^ release, mediated by inositol 1,4,5-trisphosphate receptors (IP_3_Rs), from intracellular stores in boar spermatozoa.

It is well established, and confirmed in the present study, that subsets of the heterogeneous sperm population are particularly sensitive to chilling injury, exhibiting a loss of motility and membrane integrity, especially when rapidly cooled and stored in the basic short-term semen extender used here. Surviving spermatozoa constitute a more resistant population, which, nonetheless, may experience sublethal damage. This could affect essential steps in fertilization, many of which are tightly regulated by cytosolic Ca^2+^.

After ejaculation, and before activation in the female reproductive tract, cytosolic Ca^2+^ concentrations in functionally intact spermatozoa are maintained at low levels (<100 nM; Ho, et al. [18]). Increased cytosolic Ca^2+^ concentrations in freshly ejaculated or stored semen samples are associated with membrane destabilization and premature stages of capacitation, caused by either the uptake of extracellular Ca^2+^ or release from intracellular stores [17,19]. To examine alterations in the mobilization of internal Ca^2+^ stores, in the present study, all steps of semen processing (i.e., dilution, chilling, storage, and rewarming) were performed in a Ca^2+^-free, non-capacitating environment. Baseline levels of free intracellular Ca^2+^ were lower in viable acrosome-intact spermatozoa from chilled samples (5 °C) than in the control fresh samples (day 0, RT) and in the samples stored at the conventional temperature (17 °C). In contrast, an earlier fluorometric study reported a significant increase in internal Ca^2+^ immediately after chilling boar spermatozoa in an essentially Ca^2+^-free PBS (without an added Ca^2+^ chelator), which was subsequently reversed after incubation at 25 °C for 150 min [17]. In the present study, Ca^2+^ concentrations were studied exclusively in the subpopulation of plasma membrane-intact, non-capacitated spermatozoa showing no signs of acrosomal exocytosis. It is suggested that the cell population surviving the chilling injury (approximately 50% in the present study) possesses a highly efficient internal regulatory system that allows them to maintain low cytosolic Ca^2+^ concentrations in the absence of extracellular Ca^2+^.

Storage duration exerted less stress on the Ca^2+^-regulatory system than relatively rapid cooling in a non-cold-shock protective semen extender. Baseline fluorescence intensities for the Ca^2+^ probe (before addition of thimerosal) indicate that the effects of temperature and storage on intracellular Ca^2+^ levels became less pronounced with continued rewarming of the samples to body temperature. By the end of incubation (120 min), the preceding storage temperature no longer influenced the cytosolic Ca^2+^ levels, indicating the high efficiency of internal Ca^2+^-regulatory mechanisms in restoring Ca^2+^ homeostasis. We suggest that warming reactivates ATP-dependent Ca^2+^-regulatory units, which promote Ca^2+^ uptake into intracellular stores. Calcium transport from the cytosol into stores normally occurs against the electrochemical gradient and, therefore, requires energy. Typically, this is achieved by ATPase pumps such as the sarco/endoplasmic reticulum Ca^2+^-ATPase (SERCA) and secretory pathway Ca^2+^-ATPases (SPCA). Additionally, Ca^2+^ exchangers may be involved [5]. In boar semen stored for three days at 5 or 17 °C, the ATP content and energy charge were found to be highly correlated with sperm viability, suggesting that surviving cells maintain efficient energy metabolism that allows them to reconstitute Ca^2+^ regulation. Additionally, the previous study showed that the ATP content and energy charge in sperm rewarmed to 38 °C after storage for 24 h at 5 °C did not differ from the sperm that were stored at 17 °C, indicating a high resilience of energy metabolism to chilling stress that was provoked by rapid cooling in a basic short-term semen extender [20].

Mobilization of Ca^2+^ from intracellular stores is a key event in capacitation, hyperactivation, and the acrosome reaction [5,21]. A main objective, therefore, was to investigate whether semen chilling and storage affect the function of thimerosal-sensitive intracellular Ca^2+^ stores in viable boar spermatozoa. Thimerosal stimulates Ca^2+^ flux through IP_3_Rs and RyRs in a similar manner, probably by targeting a highly conserved sequence containing two cysteine residues near the carboxyl terminus of these receptors [22]. Treatment of spermatozoa with thimerosal stimulates the release of Ca^2+^ from intracellular stores [23]. In many cell types, IP_3_Rs and ryanodine receptors (RyRs) represent the two principal intracellular Ca^2+^ channels responsible for releasing stored Ca^2+^ [12]. In boar spermatozoa, type 1 IP_3_Rs (IP_3_R1) are present in the connecting piece and the acrosome [24], and it was also reported that IP_3_R1 is located in the neck region and, at a lower density, along the axonemal membrane [11]. The presence of RyRs in boar spermatozoa remains unknown. They have been detected in mature rodents [25] and human spermatozoa [14], but not in bovine spermatozoa [12].

In the present study, the addition of thiomersal to a Ca^2+^-free, non-capacitating medium induced a dose-dependent increase in internal Ca^2+^ within seconds. Thimerosal was used at a concentration of 100 µM to illustrate the relative change in cytosolic Ca^2+^, although a lower concentration (25 µM) was also effective. Hyperactivated motility has been induced using different concentrations of thimerosal, such as 20–100 µM, in bulls [12], 25 µM in boar [26], 5 µM in humans [27], and 50 µM in mice [28,29]. In our study, the kinetics of Ca^2+^ release revealed a dose-dependent relative increase in internal Ca^2+^ over 5 min following the addition of thimerosal. Storage reduced spermatozoa’s initial response to thimerosal regardless of the temperature. However, rewarming the samples prior to a prolonged thimerosal challenge abolished the storage effect, supporting the idea of an active, energy- and/or ion flux-dependent restorative mechanism of intracellular Ca^2+^ homeostasis. Notably, hypothermic semen storage at 5 °C, compared with 17 °C, prior to rewarming, did not affect thimerosal-induced Ca^2+^ mobilization. Likewise, the level of the response to thimerosal in spermatozoa stored at 5 °C and 17 °C was similar, regardless of the duration of pre-incubation at 38 °C. Together, these observations point to an efficient and robust activation mechanism of IP_3_ receptors that enables well-controlled Ca^2+^ release in spermatozoa that have survived chilling stress. Whether chilling affects the upstream components of the phosphoinositide signaling pathway, leading to the generation of IP_3_, or interferes with the formation or function of receptor-mediated activators, was not the subject of the present study. The activity of IP_3_R- and RyR-gated channels is modulated by several factors, including Ca^2+^, magnesium, ATP, and post-translational modifications [5]. Altered mobilization of internal Ca^2+^ stores due to chilling-induced imbalances in these components cannot be ruled out. Nonetheless, the observed high resilience of viable sperm after chilling to 5 °C is in agreement with previous observations in boar semen doses that were subjected to stress from storage [30] and transport-related vibrations [31,32]. In agreement with the results of the present study, chilled sperm maintain their functional integrity for capacitation and associated mitochondrial activity [33], both of which rely on the dynamic regulation of intracellular Ca^2+^.

In conclusion, chilling and storage do not affect the function of thimerosal-sensitive intracellular Ca^2+^ channels in viable acrosome-intact boar spermatozoa and thus confirm the safety for 5 °C storage protocols with respect to Ca^2+^ regulation. From a practical perspective, semen storage at 5 °C is advantageous for a reduction in bacterial growth. The present study revealed that spermatozoa surviving the initial chilling stress maintain their ability to regulate Ca^2+^ homeostasis by intracellular stores. Furthermore, besides enabling the omission of antibiotics from the semen extender, cold-stored sperm may even offer better long-term preservation than boar semen stored at 17 °C due to a higher stress resilience in the surviving sperm population.

## 4. Materials and Methods

### 4.1. Chemicals and Reagents

Unless otherwise stated, all chemicals were obtained from Sigma-Aldrich (Steinheim, Germany), Merck (Darmstadt, Germany), and Roth (Karlsruhe, Germany). Propidium iodide (PI) and Hoechst 33342 were obtained from Axxora (Lörrach, Germany). Fluo-4/AM and PNA-Alexa Fluor^TM^ 647 were obtained from Invitrogen (Thermo Fisher Scientific, Rockford, IL, USA). Thimerosal was obtained from Sigma-Aldrich (Steinheim, Germany).

### 4.2. Semen Collection and Processing

Semen (sperm-rich and sperm-poor fractions) was collected by trained personnel from a total of six mature, clinically healthy boars that were housed at the Unit for Reproductive Medicine, University of Veterinary Medicine Hannover, Germany. The boars, aged 12 months to 5 years, belonged to the breeds Piétrain, German Large White, and crossbred animals. Housing conditions and any handling of the boars were performed in accordance with the European Commission Directive for Pig Welfare and were approved by the Institutional Animal Welfare Committee of the University of Veterinary Medicine Hannover. Semen was collected using the “gloved-hand” technique and filtered through gauze to remove the gel fraction. Immediately after collection, the semen samples were transferred to the laboratory in insulated boxes. Only normospermic ejaculates were used for the experiments, defined as ejaculates with a volume ≥ 100 mL, concentration ≥ 160 × 10^6^ sperm/mL, ≥70% motile sperm, ≤25% morphologically abnormal sperm, and ≤15% sperm with cytoplasmic droplets.

All semen samples were extended to a concentration of 20 × 10^6^ sperm/mL with the pre-warmed (32 °C) short-term extender Beltsville Thawing Solution (BTS: 176.61 mM glucose, 20.4 mM trisodium citrate dihydrate, 15.47 mM NaHCO_3_, 10.73 mM KCl, 3.49 mM Na_2_-EDTA and 260 µg/mL gentamicin sulfate (SERVA, Heidelberg, Germany); 300 ± 5 mOsmol/kg; pH 7.0 at 22 °C).

Extended semen samples were kept at room temperature (RT, 21 ± 1 °C) for 120 min (d 0), after which a subsample was analyzed, and the remainder of the samples were moved to a 17 °C storage unit. Samples designated for final storage at 5 °C were held for 120 min at 17 °C before being transferred to a 5 °C storage unit.

### 4.3. Experimental Design

In Experiment 1, a dose–response of viable intact sperm to four different concentrations of thimerosal, 0 µM (control), 25 µM, 50 µM, and 100 µM, in freshly diluted semen, was studied. In Experiment 2, the extended semen was analyzed for sperm motility, viability, acrosome integrity, and baseline free intracellular Ca^2+^ levels in viable acrosome-intact sperm. In addition, changes in free intracellular Ca^2+^ levels in viable acrosome-intact spermatozoa upon stimulation with 100 µM thimerosal after 3 min, 60 min, and 120 min incubations at 38 °C, reflecting the body temperature in the pig’s female tract, were investigated. Analysis was performed on semen stored at 17 °C and 5 °C at four different time points after dilution: at 2 h (d 0), 24 h (d 1), 72 h (d 3), and 120 h (d 5).

### 4.4. Assessment of Sperm Motility

Diluted semen samples were incubated for 30 min at 38 °C in a water bath and assessed by computer-assisted sperm analysis (CASA) using AndroVision version 9.1 (Minitube, Tiefenbach, Germany), as described in Henning et al. [34]. Five successive fields in the central axis of a Leja chamber (Leja Products B.V., Nieuw-Vennep, The Netherlands) were recorded as a video (0.8 s, 60 frames per second). The AndroVision software considered sperm to be motile when their amplitude of lateral head displacement (ALH) exceeded 1.0 µm, and their curvilinear velocity (VCL) exceeded 24.0 µm/s.

### 4.5. Sample Incubation and Flow Cytometry

Aliquots of diluted semen were incubated with 2 µM of the Ca^2+^-sensitive probe Fluo-4/AM and 0.375 μg/mL Hoechst 33342 for 30 min at RT. Subsequently, 10 µL of stained sperm were added to 1990 µL of pre-warmed (38 °C), Ca^2+^-free Tyrode’s medium that was supplemented with Hoechst 33342 (final concentration: 0.6 µg/mL), PI (2 µg/mL), and PNA-Alexa Fluor™ 647 (3 µg/mL). The Ca^2+^-free Tyrode’s medium consisted of 112 mM NaCl, 3.1 mM KCl, 1 mM Na_2_-EGTA, 0.4 mM MgSO_4_, 5 mM glucose, 0.3 mM KH_2_PO_4_, 20 mM HEPES, 21.6 mM sodium lactate, 1 mM sodium pyruvate, 3 mg/mL of bovine serum albumin (BSA; Cohn’s Fraction V, fatty acid free, Sigma-Aldrich, Steinheim, Germany), 100 µg/mL of gentamicin sulfate and 20 µg/mL of phenol red with a final osmolality of 300 ± 5 mOsmol/kg. The pH was adjusted to 7.4 at 38 °C using NaOH. Subsequently, the samples were incubated in a metal heating block at 38 °C for 3 min, 60 min, or 120 min, respectively. After each incubation time, the samples were assessed on a CytoFlex flow cytometer (Beckman Coulter, Krefeld, Germany) that was controlled by “CytExpert” software (version 2.3, Beckman Coulter). Laser lines for excitation were at 405 nm (80 mW), 488 nm (50 mW), and 638 nm (50 mW), respectively. Emission filters for the detection of blue fluorescence (450/45 nm; Hoechst 33342), green fluorescence (525/40 nm, Fluo-4), orange fluorescence (585/42 nm; propidium iodide), and red fluorescence (660/10 nm; PNA-Alexa Fluor^TM^ 647) were used. A silicone tubing (length: 12 cm; inner diameter: 0.3 mm; order no. 14164, Reichelt Chemietechnik, Heidelberg, Germany) was placed on the sample pick-up probe and inserted into the sample tube. This setup made it possible to keep the sample tube in the metal heating block at 38 °C during the assessment and add compounds at designated time points.

The samples were read at a flow rate of 100 to 200 events per second for a total period of six minutes (360 s). The first 30 s of the recording served to assess the baseline values for the fluorescence intensity of Fluo-4 in viable acrosome-intact single sperm before thimerosal at a final concentration of 25 µM, 50 µM, or 100 µM was added to the sample. Control samples were run with the addition of the solvent only, i.e., distilled water. Data from the second 10 to 30 of the baseline reading were used to assess the percentage of viable acrosome-intact sperm as a basic measure of sperm quality.

The gating strategy started by defining a logical gate for DNA-containing events (Hoechst 33342-positive) with a forward scatter signal in the size range of single sperm. By this, debris and agglutinated sperm were excluded from the evaluation. Next, the population of viable sperm with intact acrosomes (PI-negative and PNA-Alexa Fluor^TM^ 647-negative) was defined. An overlap of emission spectra for PI and PNA-Alexa Fluor^TM^ 647 was corrected after the acquisition by mathematical compensation. Values for the Fluo-4 fluorescence intensity in viable acrosome-intact single sperm were exported to an Excel file, and the average free intracellular Ca^2+^ concentration, i.e., Fluo-4 fluorescence intensity, for viable acrosome-intact single sperm was calculated by Kaluza software (Version 2.1, Beckman Coulter) for each second of the measurement.

**Figure 5:**
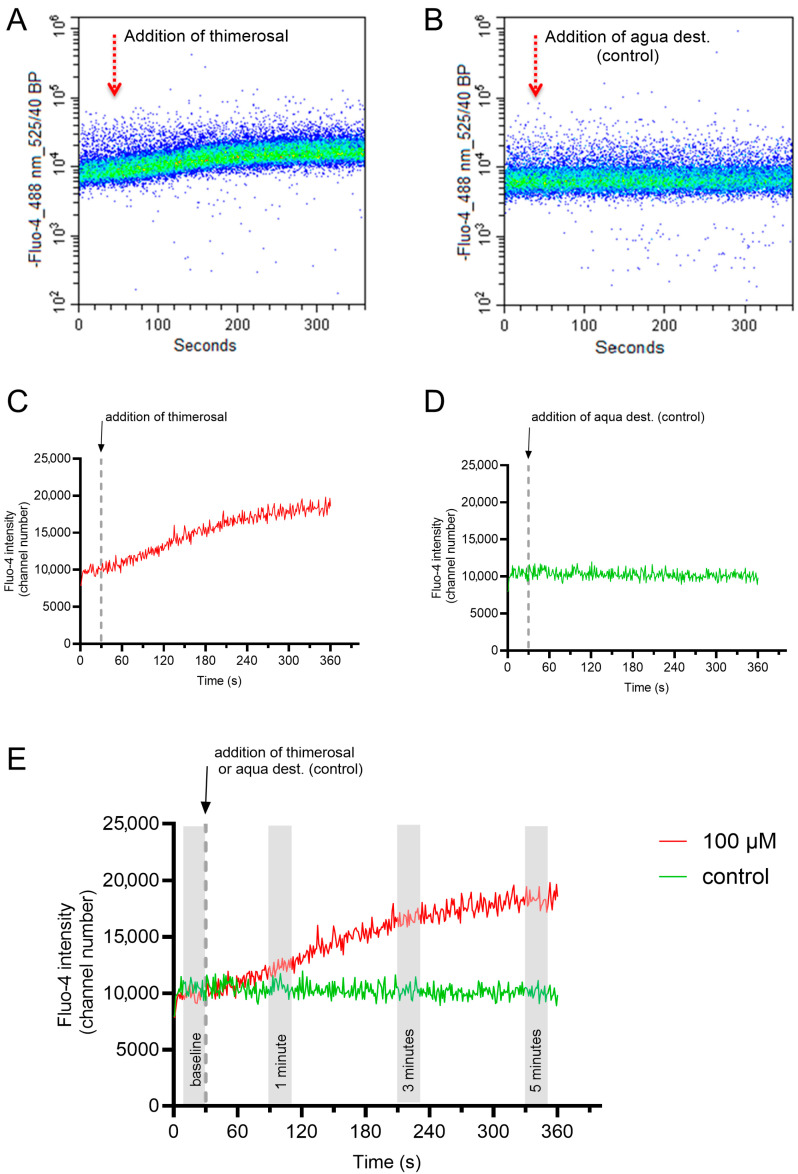
Representative flow cytometry plot for the changes in the free intracellular Ca^2+^ concentration in viable acrosome-intact sperm upon addition of 100 µM thimerosal or the solvent (aqua dest; control). Data were gated to restrict the analysis to single, viable, and acrosome-intact sperm (Hoechst 33342-positive, PI-negative, and PNA-Alexa Fluor^TM^ 647-negative). The flow cytometry plots show the change in fluorescence intensity for Fluo-4 based on the single sperm data during the assessment (**A**,**B**). The corresponding graph depicts the average change in fluorescence intensity for each second of the assessment (**C**,**D**). For systematic comparisons of the response curves, fluorescence intensities over 20 s (Seconds 10 to 30) were averaged to represent the baseline Ca^2+^ values, as well as the Ca^2+^ levels at 1 min (Seconds 80 to 100), 3 min (Seconds 200 to 220), and 5 min (Seconds 320 to 340) after the addition of thimerosal/aqua dest were determined (**E**).

A representative flow cytometry plot for the changes in the free intracellular Ca^2+^ concentration in viable acrosome-intact single sperm upon addition of thimerosal or aqua dest. are given in Figure 5A,B. Each dot in the density plots represents a single spermatozoon with its fluorescence intensity for Fluo-4 (y-axis) at a given time (x-axis). The fluorescence intensities of 100 to 200 spermatozoa per second were averaged and plotted in Figure 5C,D. The zig-zag pattern corresponds to slight variations in the average fluorescence intensity of the analyzed subsample of sperm at each second. In order to minimize a putative “sampling bias” on the interpretation of the data, intracellular Ca^2+^ levels of spermatozoa were determined by averaging the Fluo-4 fluorescence intensity for 20 s at four different periods, i.e., before (baseline) and 1, 3, and 5 min after the addition of thimerosal or aqua dest. (Figure 5E).

### 4.6. Imaging of Free Intracellular Calcium in Viable Boar Spermatozoa

Exemplary imaging of spermatozoa was performed on samples on the day of semen collection (d 0) for three boars. Aliquots of diluted semen in Ca^2+^-free Tyrodes’s medium supplemented with PI and Fluo-4/AM, as described above, were placed on pre-warmed (38 °C) glass slides, covered with pre-warmed cover slips (18 × 18 mm), and imaged at 1000× magnification with immersion oil on the heated stage (38 °C) of an Olympus BX 60 microscope (Olympus, Hamburg, Germany) with phase contrast optics, a mercury lamp, and a filter for 460–490 nm excitation and 515 nm long pass emission. Images were continuously acquired by a Gryphax NAOS color camera and Gryphax software, version 2.2.0.1234, (both Jenoptik AG, Jena, Germany) at 30 frames per second in fluorescence mode and saved as mpeg4 files. Individual frames were extracted from the video files for analysis using ImageJ, version 1.48v [35]. Frames were chosen, in which the entire head and anterior midpiece were in focus. Color channels (RGB) were separated, and the gray-scale image corresponding to the green channel was analyzed. A segmented line was drawn in the central axis of the sperm head and tail. Gray values for each pixel along the line were determined by ImageJ and exported for further processing.

### 4.7. Statistical Analysis

Data were analyzed using the statistical package SAS Enterprise Guide for Windows (version 7.1, SAS Institute Inc., Cary, NC, USA). Data from all assessments are presented as the mean ± SD (standard deviation). Data were tested for normal distribution with the Shapiro–Wilk test (PROC UNIVARIATE), and for equality of variances, with Mauchly’s test for sphericity. One-way and two-way repeated-measure ANOVAs were performed to assess the effects of storage temperature, storage time, and thimerosal concentration on the changes in intracellular Ca^2+^ concentration at defined time points after the thimerosal addition. The global test was followed by either two-sided Dunnett *t*-tests to identify after which time of storage the response differed from the response recorded in fresh diluted samples, or by a paired Student’s *t*-test to compare the samples stored at 5 °C and 17 °C, or the samples exposed to different thimerosal concentrations. The probability value of *p* < 0.05 was considered statistically significant.

## Figures and Tables

**Table 1:** Motility characteristics and viability for boar spermatozoa at the day of semen dilution (Day 0) and during storage at 17 °C or 5 °C for up to five days. All data are the means and standard deviations (mean ± SD). Different letters (a,b) within a column indicate significant differences (*p* < 0.05; n = 6 boars).

		Computer-Assisted Semen Analysis						Flow Cytometry
Storage		Total Motility (%)	VCL (µm/s)	VSL (µm/s)	LIN	ALH (µm)	BCF (Hz)	Viable Acrosome-Intact (%)
Day 0,	RT	88.4 ± 2.5 a	135.8 ± 24.9	71.9 ± 8.8 a	0.54 ± 0.06 a	1.03 ± 0.20	33.1 ± 2.0	80.7 ± 1.3 a
Day 1,	17 °C	88.2 ± 2.8 a	128.4 ± 23.1	65.4 ± 9.2 ab	0.52 ± 0.05 ab	0.99 ± 0.17	33.4 ± 3.3	81.6 ± 1.4 a
5 °C		65.3 ± 10.8 b	123.6 ± 22.3	61.4 ± 7.0 ab	0.52 ± 0.09 ab	0.95 ± 0.18	31.3 ± 4.1	61.8 ± 6.7 b
Day 3,	17 °C	87.1 ± 2.5 a	131.7 ± 23.1	69.8 ± 5.6 ab	0.54 ± 0.08 a	1.00 ± 0.17	33.3 ± 5.0	81.7 ± 2.5 a
5 °C		61.2 ± 6.6 b	146.3 ± 26.0	61.2 ± 2.5 ab	0.43 ± 0.09 ab	1.15 ± 0.23	32.0 ± 3.4	66.4 ± 5.6 b
Day 5,	17 °C	84.6 ± 3.3 b	129.4 ± 24.6	66.5 ± 8.7 ab	0.52 ± 0.06 ab	0.99 ± 0.19	32.3 ± 3.7	81.6 ± 2.0 a
5 °C		61.4 ± 9.4 b	157.2 ± 30.5	59.5 ± 2.7 b	0.39 ± 0.10 b	1.27 ± 0.28	31.2 ± 3.6	62.8 ± 4.6 b

VCL: curvilinear velocity; VSL: straight line velocity; LIN: linearity (VSL/VCL); ALH: amplitude of lateral head displacement; BCF: beat-cross frequency; viable acrosome-intact: propidium iodide-negative and PNA-Alexa Fluor^TM^ 647-negative sperm.

## Data Availability

The original contributions presented in this study are included in the article and Supplementary Materials. Further inquiries can be directed to the corresponding author.

## Supplementary Materials

The following supporting information can be downloaded at: https://www.mdpi.com/article/10.3390/ijms27031248/s1.
