# Supplementary material for: Chilling Does Not Affect the Functionality of Intracellular Calcium Stores in Viable Boar Sperm During Liquid Preservation
Source: Int J Mol Sci. 2026 Jan 27;27(3):1248. doi: 10.3390/ijms27031248 (PMC12898854; doi:10.3390/ijms27031248)
Supplement: Supplementary file 1 [file ijms-27-01248-s001.zip › Suppl. Fig. 2.pdf]

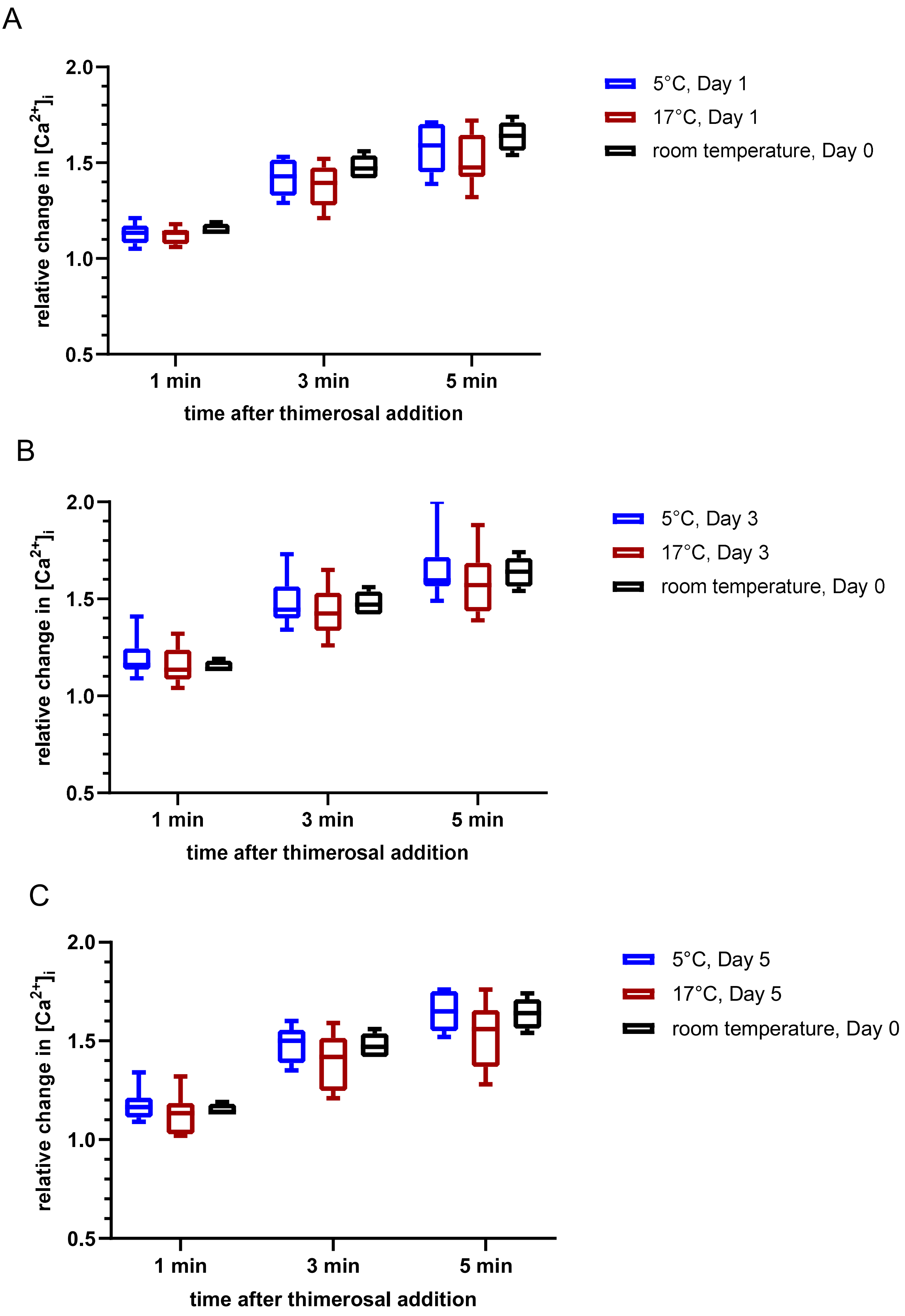

Supplemental Figure 2

Relative change in averaged free intracellular Ca^{2+} level of viable, acrosome intact spermatozoa after 60 min incubation at 38 °C in Tyrode's medium (1 mM EGTA, no Ca^{2+} added) subsequent exposure to thimerosal (100 µM). Changes are presented for readings at 1, 3 and 5 minutes after addition of thimerosal. Changes in samples at room temperature at the day of semen collection (black symbols) is compared to samples stored at 17°C or 5°C for one (A), three (B), or five (C) days (n = 6 boars). Changes in fluorescence intensity for Fluo-4 (F1) in relation to a reference point (F0), i.e., the baseline fluorescence intensity before addition of thimerosal, have been calculated (relative intracellular Ca^{2+}-level = F1/F0).
